# Longitudinal vibration compensation model of stepped-pipe strings in deep-sea mining

**DOI:** 10.1371/journal.pone.0241650

**Published:** 2020-11-09

**Authors:** QingHui Song, HaiYan Jiang, QingJun Song, Linjing Xiao, Qiang Liu

**Affiliations:** 1 Department of Mechanical and Electronic Engineering, Shandong University of Science & Technology, Qingdao, Shandong, China; 2 Tai-an School, Shandong University of Science & Technology, Tai-an, Shandong, China; University of Genova, ITALY

## Abstract

In this paper, the dynamic model of the rigid space stepped-pipe strings system is derived with Lagrangian method to represent the system dynamic behaviors which enriches the analysis method of longitudinal vibration of stepped-pipe strings. The stepped-pipe strings is constructed of pipes with different diameters and lengths, the physical properties of which mainly depends on the axial force and the depth of deep-sea mining. Based on lumped element method, the heave compensation system with dynamic vibration absorber is designed for longitudinal vibration suppression of the stepped-pipe strings. The analytical solution is obtained by modal analysis method when the mining ship is subjected to sea breeze excitation. The proposed method is easily implementable for rigid space stepped-pipe strings system with complex multi-degree-of-free deep-sea mining dynamic model. Furthermore, the optimal combination of mass ratio, spring coefficient and damping ratio is shown to have a better vibration suppression performance. Finally, numerical simulations on the stepped-pipe strings system with or without dynamic vibration absorbers are provided to demonstrate the effectiveness of the proposed method.

## 1 Introduction

With the deceasing of the mineral resources in the land, the exploitation and use of seafloor poly-metallic mineral resources have attracted more and more attention of the global mining industry [1], and the metals in these mineral are essential for various high-tech, green-tech, emerging-tech, and energy applications [2], so the deep-sea mining (DSM) has become an important research subject. A promising DSM system has been proposed which is an integration of an ore collection system, a mining ship and a transportation system. The transportation system is an important part of deep-sea mining system, which transports the collected Ferromanganese (Fe-Mn) crusts from the miners to the mining ship by pump-lift method [3]. However, the stepped-pipe strings is easy to vibrate longitudinally, laterally and torsionally due to the motions of ship and waves, of which the longitudinal vibration is the most critical [4]. Aso, et al. indicated that the force more than 3.03xl0^6^N was required to control the longitudinal vibration of the pipe string if the active control was applied on the ship, it is difficult to obtain a lot of power on the ship or the stepped-pipe strings [5].

Given the above context, many researchers in the DSM field began to study the vibration reduction of stepped-pipe strings.Because the dynamic vibration absorber (DVA) is simple and economical, this system has potential advantages over other types of vibration control devices in suppressing the vibration [6]. Aso, et al. proposed a semi-active control, the buffer's vibration absorber with variable spring constant according to the ship' heave-motion was studied to reduce the longitudinal vibration [5]. Based on the studies, a vibration absorber was attached to the pump module as well as the buffer to reduce the longitudinal vibration of the pipe strings for mining the cobalt crust at the bottom of the deep sea, the absorber attached to the buffer causes much greater effect on the vibration of the pipe strings than that attached to the pump module [7]. In the literature [8], the pump-lift system with the pipe string equipped with two pump modules instead of one pump module in Ref. [7], and gave an optimum combination of the mass and spring constant for the absorber. Applying the method of separation of variables, Erol studied the over damping and under damping modes of the hoisting system respectively, and obtained the exact analytical solutions of free response and forced response of the system damping under the heave motion of the mining ship [9].Taking the minimum of the displacement variance of the proposed heave compensation system as the optimal object and considering the allowed displacement of the dynamic vibration absorber itself, the computation formula of the optimum parameters of dynamic vibration absorber for heave compensation system of deep-sea mining was derived [10]. Tang et al. set up the vibration test system of a stepped-pipe strings with a manganese nodules pump in lab, the vibration characteristics of the pipe were measured and analyzed, and found that the dynamic load is much higher than static load [11]. Liu and Xiao studied the longitudinal vibration characteristics of the 5000m mining pipe in the sea under different working wind conditions, offset angle, damping, and ore bin weight [12]. Yang et al. set up the heave compensation model combined with a vibration absorber and accumulator according to the requirements of 6000 meter sea for poly-metallic nodule mining systems in China [13].

All of the aforementioned vibration absorbers applied Newtonian mechanics to establish the differential equation of the system, which often introduces some unknown binding force, so it is more complicated for complex system. In fact, the lifting is a coupled complex system, and it is difficult to study the interaction between ship and slender components in deep water operation by model test owing to the limitation of pool depth [14]. Therefore, it was meaningful to study a simple and convenient modeling method to meet the needs of longitudinal vibration analysis in the lifting system.

In order to predict the dynamic behavior of this integrated mining system,the dynamics simulations must be carried out before the experimental design verification begins [15]. Therefore, computer simulation based design is required to analyze dynamic performance of stepped-pipe strings for the design process.When compared with aforementioned vibration absorbers on the longitudinal vibration of stepped-pipe strings in the DSM system, the dynamic vibration absorber proposed in this paper is different in the following aspects: 1)The Lagrangian equation is used to establish the motion equation of the longitudinal vibration system, which has obvious advantages for the complex coupling stepped-pipe strings system; 2)Analyze the principal vibration mode of stepped-pipe strings under level-6 sea conditions, the deformation characteristics and the main deformation positions are obtained, which provides a basis for the design of the dynamic vibration absorber; 3) attach dynamic vibration absorbers to the stepped pipes with large longitudinal vibration amplitude, and carry out the modal simulation; 4) detailed analytical results are presented to guarantee the dynamic performance improvement of vibration absorber, even in the presence of parameter coupling variation.

The rest of the paper is organized as follows. Section 2 briefly reviews the stepped-pipe strings for DSM and mass lumping scheme. Section 3 introduces the motion equation of stepped-pipe strings with attaching DVA, inherent characteristics of the system and modal analysis. The numerical simulations are given in Section 4, and Section 5 concludes the paper.

## 2 Mass lumping scheme of stepped-pipe strings

**Fig 1:**
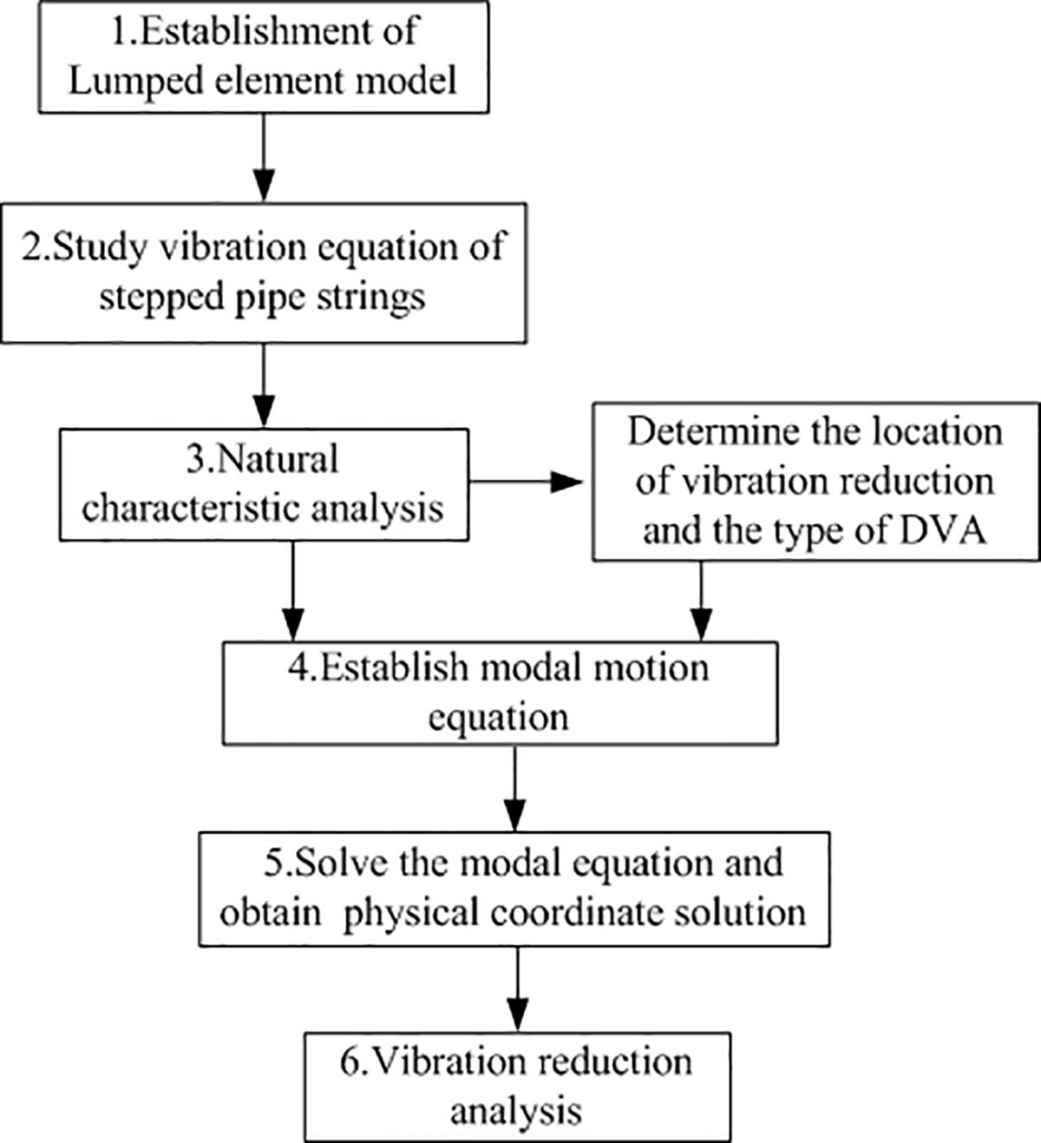
Main steps proposed to analyze the longitudinal vibration of stepped-pipe strings in DSM.

A method for longitudinal vibration analysis of stepped-pipe strings based on Lagrangian theory and mass lumping scheme was developed and applied. It consists of six main steps as shown in the flowchart in Fig 1.

### 2.1 Problem preliminaries

**Fig 2:**
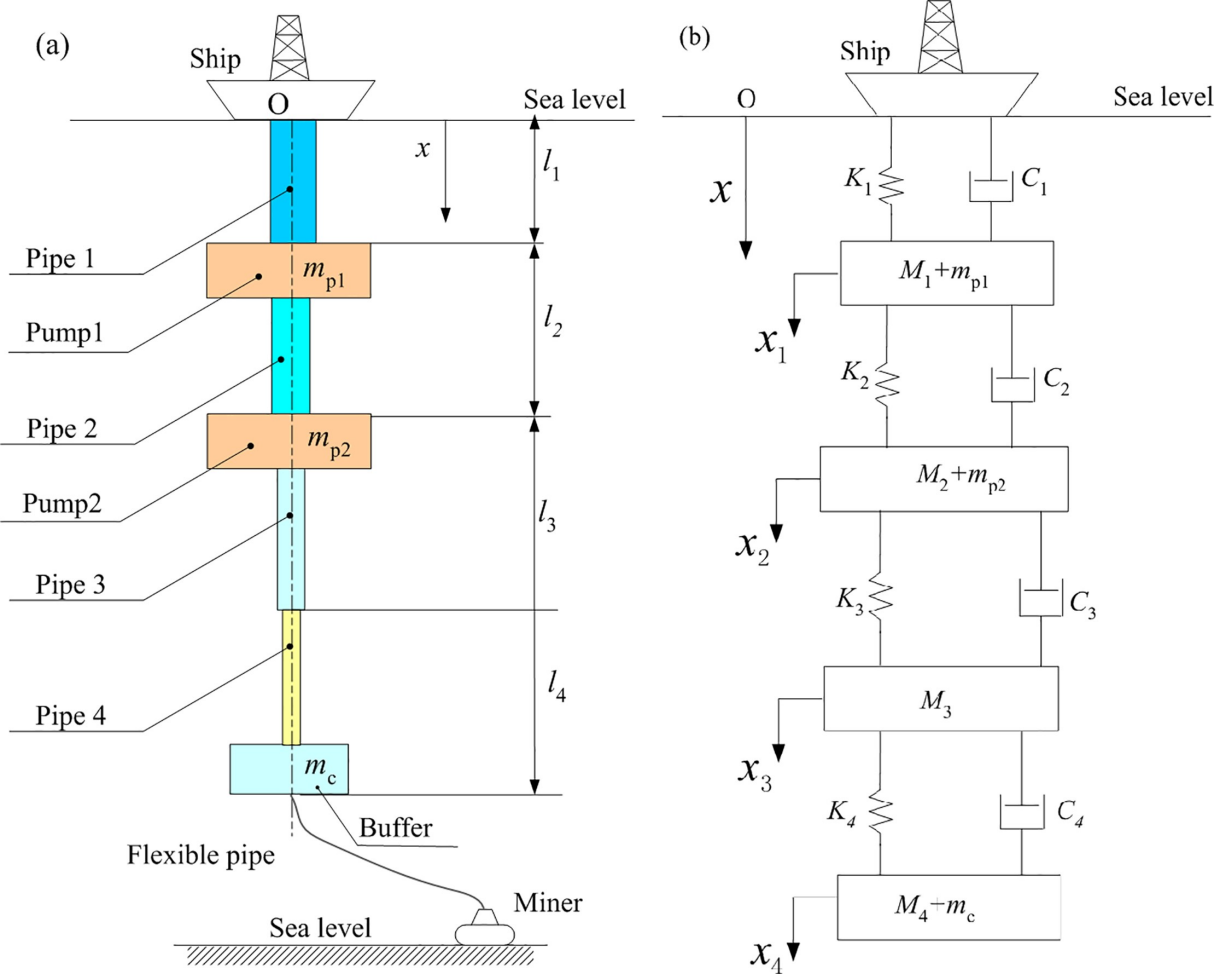
Schematic view of the model of the stepped-pipe strings. (a) is the typical structure diagram of DSM system. (b) represents the schematic view of lumped mass model of stepped-pipe strings, the stepped-pipe strings are considered as a set of four blocks (M_*i*_) that are connected to four springs with spring constants K_*i*_ and four damping elements with viscous damping coefficient *C*_*i*_.

Fe-Mn crusts are found typically at water depths of 400–7000 m, the 5000m deep-sea Fe-Mn crusts mining system is an integration of a mining ship, stepped-pipe strings, lifting pumps, flexible pipe and a seafloor miner as indicated in Fig 2(A). The stepped-pipe strings can be regarded as a four-stage step pipe, *i*-th of which has the length *l*_i_. The pump modules with concentrated masses *m*_p1_ and *m*_p2_ is installed at the end of *l*_1_ and *l*_2_ respectively, and a buffer module is installed at the end of *l*_4_ with a concentrated mass *m_c_*. It is assumed that the mining ship moves in the harmonic heave motion, the fluid inside the stepped-pipe strings vibrates with pipe, and the flexible pipe does not affect the vibration of the stepped-pipe strings.

In the aforementioned stepped-pipe strings, only the pumps and buffer are regarded as the concentrated masses, and the exact analytical solutions of the system subjected to heave motion of the mining ship are determined by the differential equations. Newton method has clear physical concept and simple method, but it is difficult to solve complex system. Because the structure of the stepped-pipe strings is characterized by the connection of several pipe sections with similar structure and high stiffness, the stepped pipes can be simplified as concentrated masses. In the dynamic analysis of practical engineering vibration system, a lumped mass matrix is always better than the consistent mass matrix, if a lumped mass matrix is employed, the solving of large scale simultaneous algebraic equations can be avoided [16]. In order to study the longitudinal coupling vibration of the lifting system more accurately, a mass lumping scheme which is suitable for the vibration system of stepped-pipe strings is proposed.

Using the idea of separation, the stepped-pipe strings is modeled as a set of four blocks that are connected sequentially by four spring elements (*K_i,_i* = 1,2,3,4), four damping elements (*C_i,_i* = 1,2,3,4) and four lumped masses (M*_i,_i* = 1,2,3,4). It is assumed that the first pump and the first pipe are considered as a concentrated mass, the second pump and the second pipe are considered as a concentrated mass, and the buffer and the fourth pipe are regarded as a concentrated mass, see Fig 2(B). The upper end of the first section is connected with the mining ship, and the bottom of the last section is connected with the flexible pipe. The position where the pipe diameter changes is the connection point, and the mass of each block is concentrated on the center of gravity of the connection point.

According to the principle of equal kinetic energy before and after simplification, the masses of the original pumps and buffer are respectively concentrated on the corresponding stepped pipes, so that the concentrated mass of the first, the second and the fourth section are respectively *M*_1_+*m*_*p*1_, *M*_2_+*m*_*p*2_ and *M*_4_+*m*_*c*_.

The every concentrated mass of stepped-pipe can be defined as

$$M_{i}=\rho_{i}l_{i}\;i=1,2,3,4$$ (1)

where *l*_*i*_ is the length of stepped-pipe, *ρ*_*i*_ is the linear density of stepped-pipe and defined as

$$\rho_{i}=\frac{\pi (D_{i}^{2}-p_{i}^{2})\rho_{p}}{4}-\frac{\pi D_{i}^{2}\rho_{w}}{4},\;i=1,2,3,4$$ (2)

whereis *ρ*_*p*_ the density of stepped-pipe, *ρ*_*w*_ is the density of sea water, *D*_*i*_ and *p*_*i*_ are the outside diameter and the inside diameter of the stepped-pipe, respectively.

Due to the complexity and variability of the marine environment and the heave and swing motion of the mining ship, the longitudinal vibration of the stepped-pipe strings is produced, which bring forward a great challenge to the stable operation of the lifting subsystem. The study of the dynamic characteristics of the stepped-pipe strings is the basis of vibration suppression. The Lagrangian method establishes differential equations based on kinetic energy and potential energy in the generalized coordinate system and therefore it has good mathematical properties and is very suitable for theoretical analysis [17].

The stepped-pipe string is vertical and a one-dimensional coordinate system is introduced, where the origin is located on the mining ship and the positive *x* is downwards. We use generalized coordinates *x*_*i*_(*t*), where *t* is time, to uniquely determine the physical state of stepped-pipe strings at any time.

### 2.2 Modal analysis of stepped-pipe strings

#### 2.2.1 Comparison with the Newton-Euler method

When the Newton-Euler method [4,5,7–9] is used to establish the equation of motion of stepped-pipe strings, the equations describing each stepped-pipe contain the coupling force because each pipe is coupled with other pipes. So, it is necessary to determine all coupling terms by forward-backward recursion, and then obtain the overall description of the stepped-pipe strings. The Lagrangian's method establishes differential equations of motion from the point of view of energy. Although the two methods take different routes, the results are exactly the same [18].

In any mechanical system, a set of generalized coordinates and a set of corresponding generalized forces can be confirmed. Dynamic analysis of a system means seeking the relationship between generalized forces and generalized coordinates. In this case, two aspects need to be distinguished as the following [19]:

1. Obtain the closed-form equations to describe the change of generalized coordinates with time.
2. Discover the generalized force to makes the generalized coordinate realize a specific time evolution trajectory.

To some extent, Lagrangian's method has advantages in the former type of analysis and Newton-Euler method has advantages on the latter case [18,19].The longitudinal vibration of a stepped-pipe strings is a complex system, belonging to the former type of analysis. Therefore, the Lagrange's method has advantages in establishing the multi-degree-of-free motion equations for the system with accurate and powerful advantages.

#### 2.2.2 Lagrangian equation

It is assumed that all the cross-sections remain plane during the longitudinal vibration of the steeped-pipe strings, and the transverse deformation caused by the longitudinal vibration is ignored, and the fluid density in the pipe is uniformly distributed. According to the Lagrangian theory, the motion equations of the stepped-pipe system can be formulated as follows based on the Lagrangian equation:

$$\frac{d}{dt}\left(\frac{\partial T}{\partial {\dot{x}}_{i}}\right)-\frac{\partial T}{\partial x_{i}}+\frac{\partial V}{\partial x_{i}}+\frac{\partial D}{\partial {\dot{x}}_{i}}=Q_{i},i=1,2,\ldots 4$$ (3)

where *T*,*V* and *D* are the kinetic energy, potential energy and energy dissipation functions of the system, respectively, *Q*_*i*_ is the generalized driving force, *x*_*i*_ is the generalized coordinate, {*x*(*t*)} = {*x*_1_(*t*) *x*_2_(*t*) *x*_3_(*t*) *x*_4_(*t*)}^*T*^.

The total kinetic energy of the stepped-pipe system is

$$T=\frac{1}{2}\left(M_{c1}{\dot{x}}_{1}^{2}+M_{c2}{\dot{x}}_{2}^{2}+M_{c3}{\dot{x}}_{3}^{2}+M_{c4}{\dot{x}}_{4}^{2}\right)$$ (4)

where *M*_*c*1_ = *M*_1_+*m*_*p*1_, *M*_*c*2_ = *M*_2_+*m*_*p*2_, *M*_*c*3_ = *M*_3_ and *M*_*c*4_ = *M*_4_+*m*_*c*_.

The total potential energy of the stepped-pipe system can be calculated as follow

$$D=\frac{1}{2}\left[C_{1}{\dot{x}}_{1}{}^{2}+C_{2}{\left({\dot{x}}_{2}-{\dot{x}}_{1}\right)}^{2}+C_{3}({\dot{x}}_{3}-{\dot{x}}_{2})1^{2}+C_{4}{\left({\dot{x}}_{4}-{\dot{x}}_{3}\right)}^{2}\right]$$ (5)

The total potential energy of the stepped-pipe system is given as

$$V=\frac{1}{2}\left[K_{1}x_{1}{}^{2}+K_{2}{\left(x_{2}-x_{1}\right)}^{2}+K_{3}{\left(x_{3}-x_{2}\right)}^{2}+K_{4}{\left(x_{4}-x_{3}\right)}^{2}\right]$$ (6)

Substituting Eqs (4–6) into Eq (3), the dynamic equation of motion for the stepped-pipe system can be written in compact closed form as

$$\left[M\right]\left\{\ddot{x}(t)\right\}+\left[C\right]\left\{\dot{x}(t)\right\}+\left[K\right]\left\{x(t)\right\}=\left\{f(t)\right\}$$ (7)

where [***M***]∈*R*^4×4^, [***C***]∈*R*^4×4^ and [***K***]∈*R*^4×4^ are mass matrix,damping matrix and stiffness matrix of the whole stepped-pipe system respectively, {*f*(*t*)} = {*f*_0_cosΩ*t* 0 0 0}^*T*^. Eq (7) is defined by the following matrices:

$$[M]=\left[\begin{array}{cccc} M_{c1} & 0 & 0 & 0\\ 0 & M_{c2} & 0 & 0\\ 0 & 0 & M_{c3} & 0\\ 0 & 0 & 0 & M_{c4} \end{array}\right],[C]=\left[\begin{array}{cccc} C_{1}+C_{2} & -C_{2} & 0 & 0\\ -C_{2} & C_{2}+C_{3} & -C_{3} & 0\\ 0 & -C_{3} & C_{3}+C_{4} & -C_{4}\\ 0 & 0 & -C_{4} & C_{4} \end{array}\right],[K]=\left[\begin{array}{cccc} K_{1}+K_{2} & -K_{2} & 0 & 0\\ -K_{2} & K_{2}+K_{3} & -K_{3} & 0\\ 0 & -K_{3} & K_{3}+K_{4} & -K_{4}\\ 0 & 0 & -K_{4} & K_{4} \end{array}\right].$$

#### 2.2.3 Modal analysis

The modal analysis primarily identifies the natural frequencies and mode shapes and has gained increasing popularity in both theoretical developments and practical applications [20]. Let us consider the actual stepped-pipe string of DSM example illustrated in Fig 2(B), its basic physical parameters are shown in Table 1. We calculated the corresponding natural frequencies and mode shapes.

**Table 1:** Basic physical parameters of stepped-pipe strings.

Stepped pipes					Bumps and buffer	
Pipe section	Length (m)	Internal diameter (m)	External diameter (mm)	Density kg/m^3^		Mass (kg)
I	1000	206	254	7850	Bump1	8000
II	1000	206	240	7850	Bump2	8000
III	1500	206	232	7850	Buffer	30000
IV	1500	206	226	7850	-	-

**Fig 3:**
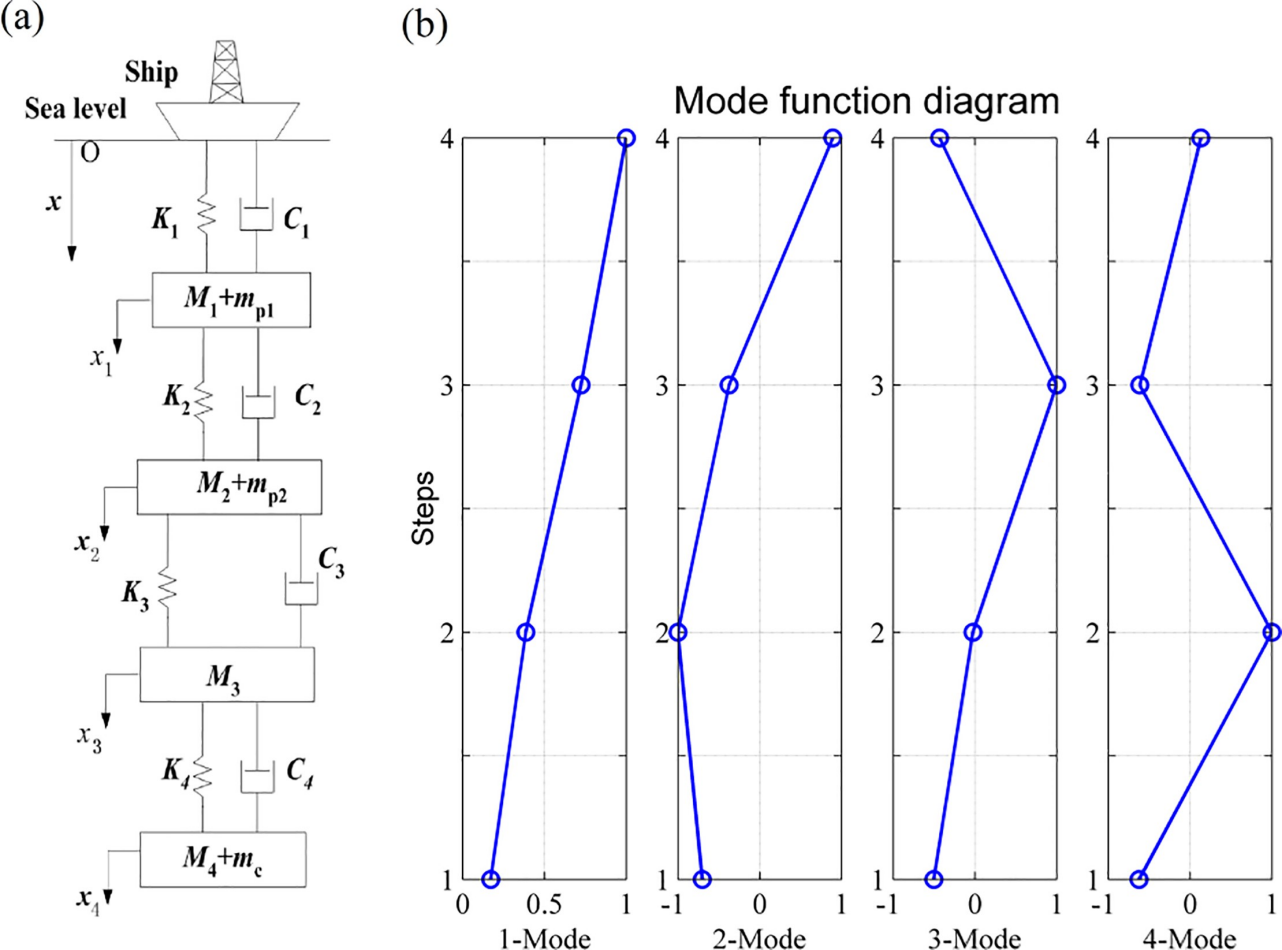
Vibration modes figures of stepped-pipe strings without DVA.

In this example, the density of the sea water is assumed to be 1024kg/m^3^, and the system has four degrees of freedom with one mode per degree of freedom. The first four orders vibration mode of stepped pipes are figured out, as shown in Fig 3.

The first four orders natural frequency and maximum deformation of stepped- pipe strings vibration is summarized in Table 2.

**Table 2:** Characteristic information of principal modes without DVA.

Modal order	Natural frequency (rad/s)	Maximum deformation (m)	Maximum deformation position
1	2.3108	5.9618	4-th stepped pipe
2	5.2270	1.8844	2-th stepped pipe
3	8.0075	0.7613	3-th stepped pipe
4	10.3957	0.3065	2-th stepped pipe

As can be seen from Fig 3 and Table 2, the maximum deformation of the first order mode is at the end of the fourth stepped pipe, and the vibration amplitude decreases from the end of the fourth stepped pipe to the beginning of the first stepped pipe. The maximum of the second order mode is at the end of the second stepped pipe, and the vibration amplitude decreases to the end of the fourth stepped pipe and the beginning of the first stepped pipe. The maximum deformation of the third order mode is at the end of the third stepped pipe, and the vibration amplitude decreases to the end of the fourth stepped pipe and the beginning of the first stepped pipe. The maximum deformation of the fourth order mode is at the end of the second stepped pipe, and the amplitudes at the end of the fourth stepped pipe and the beginning of the first stepped pipe decrease. It is thus clear that the maximum vibration amplitude point located at the end of the second and fourth stepped pipes. Then, by following the procedure outlined in Subsection 2.1, we added the DVAs at the end of the second and fourth stepped pipes, and calculated the frequencies and modes in Subsection 4.1.

## 3 Analytical investigation on stepped-pipe strings with DVA

### 3.1 Motion equation of stepped-pipe strings with attaching DVA

The DVA with damping element has always been used to suppress the amplitude of the primary vibration systems subjected to excitation in a broader frequency range [21]. When a DVA is attached to a primary system, this simple device can tremendously suppress its vibration if properly designed [22]. According to the above analysis, the vibration absorber, which is a single degree of freedom system consisting of a mass(*m*_1_,*m*_2_), spring(*k*_1_,*k*_2_) and a damper(*d*_1_,*d*_2_), is equipped at the end of the second and fourth stepped pipes, see Fig 7(A).

Let *x_i_* and *z_i_* denote the displacements of the primary system and absorber, respectively. The coordinate system is established and the generalized coordinate group (*x*_1_,*x*_2_,*z*_1_,*x*_3_,*x*_4_,*z*_2_) is selected along the vertical vibration direction, then the kinetic energy, potential energy and energy dissipation functions of the system can be expressed as:

$$\{\begin{array}{l} T=\frac{1}{2}\left(M_{c1}{\dot{x}}_{1}^{2}+m_{1}{\dot{z}}_{1}^{2}+M_{c2}{\dot{x}}_{2}^{2}+M_{c3}{\dot{x}}_{3}^{2}+M_{4}{\dot{x}}_{4}^{2}+m_{2}{\dot{z}}_{2}^{2}\right)\\ V=\frac{1}{2}\left[K_{1}x_{1}^{2}+k_{1}{\left(z_{1}-x_{1}\right)}^{2}+K_{2}{\left(x_{2}-x_{1}\right)}^{2}+K_{3}{\left(x_{3}-x_{2}\right)}^{2}+K_{4}{\left(x_{4}-x_{3}\right)}^{2}+k_{2}{\left(z_{2}-x_{4}\right)}^{2}\right]\\ D=\frac{1}{2}\left[C_{1}{\dot{x}}_{1}^{2}+d_{1}{\left(z_{1}-{\dot{x}}_{1}\right)}^{2}+C_{2}{\left({\dot{x}}_{2}-{\dot{x}}_{1}\right)}^{2}+C_{3}{\left({\dot{x}}_{3}-{\dot{x}}_{4}\right)}^{2}+C_{4}{\left({\dot{x}}_{4}-{\dot{x}}_{3}\right)}^{2}+d_{2}{\left({\dot{z}}_{2}-{\dot{x}}_{4}\right)}^{2}\right]. \end{array}$$ (8)

The equation of motion with DVA (Eq (7)) can be obtained by substituting Eq (8) into Eq (3), and Eq (7) is defined by the following matrices:

$$\left[M\right]=\left[\begin{array}{cccccc} M_{c1} & 0 & 0 & 0 & 0 & 0\\ 0 & M_{c2} & 0 & 0 & 0 & 0\\ 0 & 0 & m_{1} & 0 & 0 & 0\\ 0 & 0 & 0 & M_{c3} & 0 & 0\\ 0 & 0 & 0 & 0 & M_{c4} & 0\\ 0 & 0 & 0 & 0 & 0 & m_{2} \end{array}\right],\left[C\right]=\left[\begin{array}{cccccc} C_{1}+C_{2} & -C_{2} & 0 & 0 & 0 & 0\\ -C_{2} & C_{2}+C_{3}+d_{1} & -d_{1} & -C_{3} & 0 & 0\\ 0 & -d_{1} & d_{1} & 0 & 0 & 0\\ 0 & -C_{3} & 0 & C_{3}+C_{4} & -C_{4} & 0\\ 0 & 0 & 0 & -C_{4} & C_{4}+d_{2} & -d_{2}\\ 0 & 0 & 0 & 0 & -d_{2} & d_{2} \end{array}\right],\left[K\right]=\left[\begin{array}{cccccc} K_{1}+K_{2} & -K_{2} & 0 & 0 & 0 & 0\\ -K_{2} & K_{2}+K_{3}+k_{1} & -k_{1} & -K_{3} & 0 & 0\\ 0 & -k_{1} & k_{1} & 0 & 0 & 0\\ 0 & -K_{3} & 0 & K_{3}+K_{4} & -K_{4} & 0\\ 0 & 0 & 0 & -K_{4} & K_{4}+k_{2} & -k_{2}\\ 0 & 0 & 0 & 0 & -k_{2} & k_{2} \end{array}\right].$$

### 3.2 Inherent characteristics of the system

The heave compensation system of stepped-pipe strings for DSM based on vibration absorber is a complex system, which is composed of several subsystems, each of which has its own natural characteristics. The study of natural characteristics is helpful to analyze the cause of vibration and ensure the stability of the system.

In ideal condition, the free longitudinal vibration equation of stepped pipe is written as:

$$\left[M\right]\left\{\ddot{x}(t)\right\}+\left[K\right]\left\{x(t)\right\}=0$$ (9)

Let *ω*_*n*_ denotes the natural frequency of the system,the eigenvalue problem is expressed as:

$$([K]-\omega_{n}{}^{2}[M])\{U\}=0$$ (10)

The condition that the equation has a non-zero solution is that its coefficient determinant is equal to 0, namely, the characteristic equation of the system can be given in the following form:

$$\left|\left[K\right]-\omega_{n}{}^{2}\left[M\right]\right|=0$$ (11)

### 3.3 Modal analysis

Modal analysis is widely used to simplify, assess, and correct engineering vibration models [23]. The physical coordinates in the vibration differential equations are transformed into modal coordinates, so that the equations can be decoupled into a set of independent equations described by modal coordinates and modal parameters, then the solution of the equation is greatly simplified.

#### 3.3.1 Modal matrix

According to the concept of modal matrix and the orthogonality of modal vectors, the modal mass matrix and modal stiffness matrix can be obtained, which are denoted as

$$\left[M_{r}]={\left[U\right]}^{T}[M][U\right]$$ (12A)

$$\left[K_{r}]={\left[U\right]}^{T}[K][U\right]$$ (12B)

where [***M***_*r*_] and [***K***_*r*_] are diagonal matrices,and the *r*-order modal mass and stiffness are {***U***^(r)^}^*T*^[***M***]{***U***^(r)^} and {***U***^(r)^}^*T*^[***K***]{***U***^(r)^}, respectively.

According to Eq (11), combined with the definition of modal mass and modal stiffness, the square of r-order natural frequency is defined as

$$\omega_{nr}^{2}=\frac{K_{r}}{M_{r}}$$ (13)

In general, modal vectors generally have no weighted orthogonality to damping matrices, then Rayleigh damping model is used to diagonalize damping matrices in this paper. Rayleigh damping model was proposed by Rayleigh in1877, which is a convenient and simple uncoupling method of the equations of motion in the case of linear response [24]. Assuming that the damping matrix is a linear combination of the mass and stiffness matrix, the ideal damping ratio is obtained under two pre-selected modal frequencies. The general form of damping matrix is defined in the following manner:

$$\left[C\right]=\beta \left[M\right]+\lambda \left[K\right]=\left[\begin{array}{ccc} \beta M_{1}+\lambda K_{1} & & \\ & \ddots & \\ & & \beta M_{n}+\lambda K_{n} \end{array}\right]$$ (14)

where *β* and *λ* are the multiplier of mass matrix and stiffness matrix respectively, also called Rayleigh coefficients.

Substituting Eq (3) into (14), The damping matrix (13) can be rewritten as:

$$[C]=\left[\begin{array}{ccc} (\beta +\lambda \omega_{n1}^{2})M_{1} & & \\ & \ddots & \\ & & (\beta +\lambda \omega_{nn}^{2})M_{n} \end{array}\right]$$ (15)

Thus, the *r*-order damping ratio can be deduced

$$\xi_{r}=\frac{\beta +\lambda \omega_{nr}}{2\omega_{nr}}$$ (16)

According to the reliable test data estimation and calculated natural frequency, the two appropriate damping ratios(*i*- and *j*-order)can be obtained,then the *β* and *λ* can be calculated as:

$$\alpha =\frac{2\omega_{ni}\omega_{nj}(\xi_{i}\omega_{nj}‐\xi_{j}\omega_{ni})}{\omega^{2}{}_{nj}‐\omega^{2}{}_{ni}}$$ (17A)

$$\gamma =\frac{2(\xi_{j}\omega_{nj}‐\xi_{i}\omega_{ni})}{\omega^{2}{}_{nj}‐\omega^{2}{}_{ni}}$$ (17B)

#### 3.3.2 Normalized modal equation

In order to simplify the modal analysis, the principal coordinate {*q*} is often regularized to the normalized principal coordinate {*q*_N_}. The modal mass matrix obtained by regularization is identity matrix [**I**]. Since the diagonal element values of the modal mass matrix are usually not equal, it is necessary to select an appropriate factor for each main mode to make it equal to 1. This factor is called the normalized factor of the *r*-order, which is defined as:

$$\alpha_{r}=\frac{1}{\sqrt{M_{r}}}=\frac{1}{\sqrt{{\left\{u^{\left(r\right)}\right\}}^{T}[M]\{u^{\left(r\right)}\}}}$$ (18)

The previous modal matrix becomes the normalized modal matrix, the expression is written as:

$$\left[U_{N}\right]=[\alpha_{1}\{U^{\left(1\right)}\},\alpha_{2}\{U^{\left(2\right)}\},\ldots ,\alpha_{n}\{U^{\left(n\right)}\}]=\left[U\right]\left[\alpha_{r}\right]$$ (19)

In the same way, the normalized modal stiffness matrix and the normalized modal damping matrix can be obtained as follows

$$\left[K_{N}{}_{r}\right]={\left[U_{N}\right]}^{T}\left[K\right]\left[U_{N}\right]=\left[\begin{array}{ccc} \omega_{n1} & & \\ & \ddots & \\ & & \omega_{nn} \end{array}\right]$$ (20)

$$\left[C_{N}{}_{r}\right]={\left[U_{N}\right]}^{T}\left[C\right]\left[U_{N}\right]=\left[\begin{array}{ccc} 2\xi_{1}\omega_{n1} & & \\ & \ddots & \\ & & 2\xi_{n}\omega_{nn} \end{array}\right]$$ (21)

The physical coordinates of the system are transformed by the normalized modal matrix [***U***_N_], which can be expressed as:

$$\left\{x(t)\right\}=\left[U_{N}\right]\left\{q_{N}(t)\right\}$$ (22)

Substituting Eqs (22) into (7), the normalized modal equation can be obtained as follows:

$$\left\{{\ddot{q}}_{N}(t)\right\}+\left[2\xi_{r}\omega_{nr}\right]\left\{{\dot{q}}_{N}(t)\right\}+\left[\omega_{nr}^{2}\right]\left\{q_{N}(t)\right\}=\left\{N(t)\right\}$$ (23)

where ***N***(*t*) = ***U***_*N*_^*T*^***f***(*t*) is the normalized excitation corresponding to the generalized coordinates.

Modal equations are all decoupled, and each equation is an independent equation with corresponding modal coordinates, which is completely equivalent to a single degree of freedom. Therefore, no matter what the initial conditions are, the modal equation can be solved according to the theory of single degree of freedom.

## 4 Simulation studies

In this section, numerical simulations on a stepped-pipe strings with DVA in harmonic excitation are carried out to demonstrate the effectiveness of the proposed lumped parameter modal. The stepped-pipe strings model combined with two DVAs is shown in Fig 7(A).

### 4.1 Parameter selection rules for the system

The development of explicit optimal absorber parameters is challenging for a damped structural system since the fixed points no longer exist in the frequency response curve [6]. The longitudinal vibration of stepped-pipe strings was analyzed qualitatively by changing the parameters of DVA.

**Fig 4:**
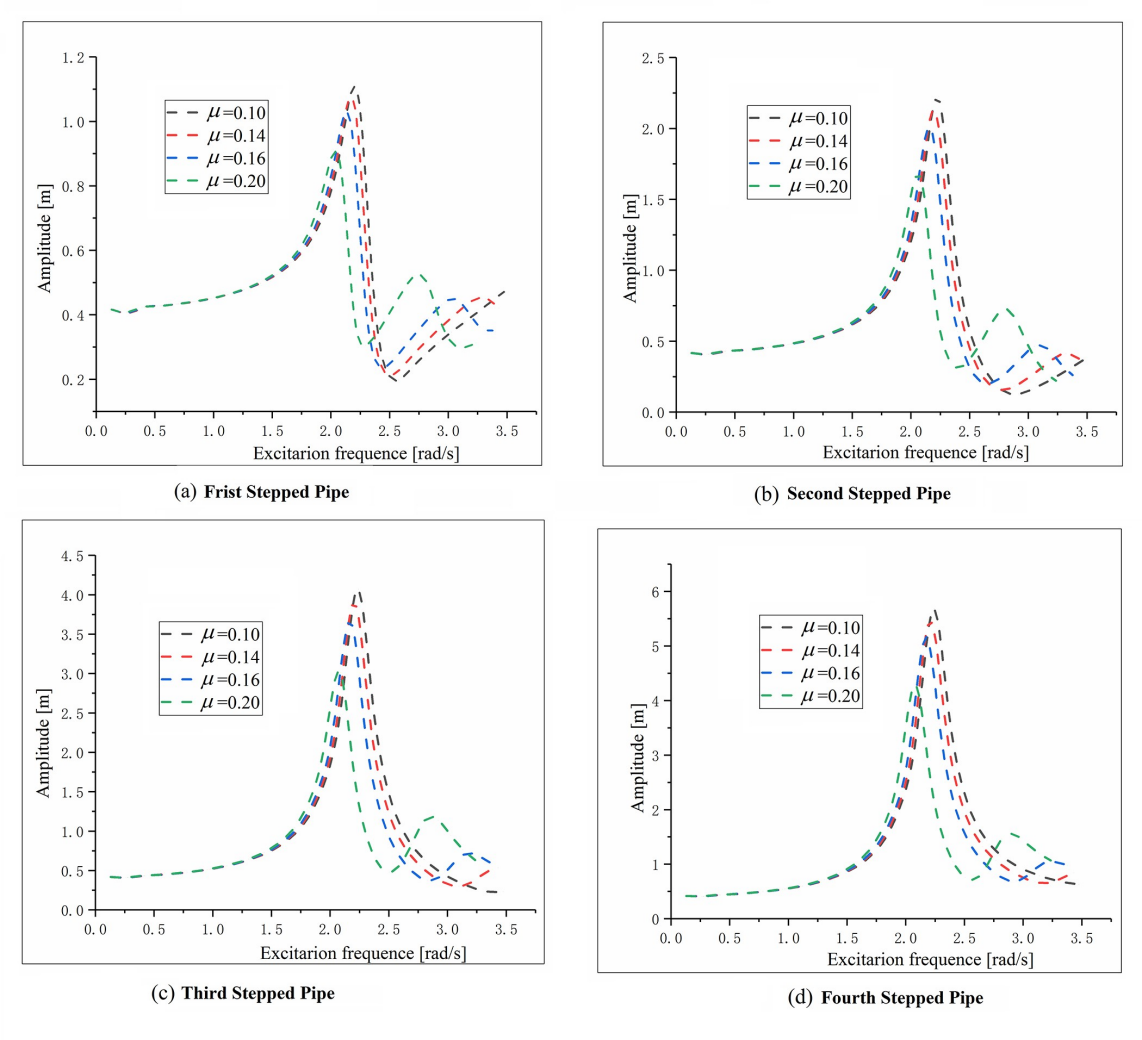
Comparison of amplitude of stepped-pipe strings for different *μ* values.

Let μ1=m1mp2, μ2=m2mc, the amplitude-frequency curves with several different *μ* values are given in Fig 4 to investigate the system characteristics.

It is clearly shown that the amplitude of each stepped-pipe is decreased with the increase of the *μ* value that the damping effect is getting better as the mass of DVA increases. However, the larger mass of DVA increases the axial stress of the stepped-pipe and the dynamic requirements of the mining ship. Generally, the mass ratio *μ* depends on the specific engineering requirement, the value range of *μ*_1_ or *μ*_2_ is within [0.1,0.2] in the system.

**Fig 5:**
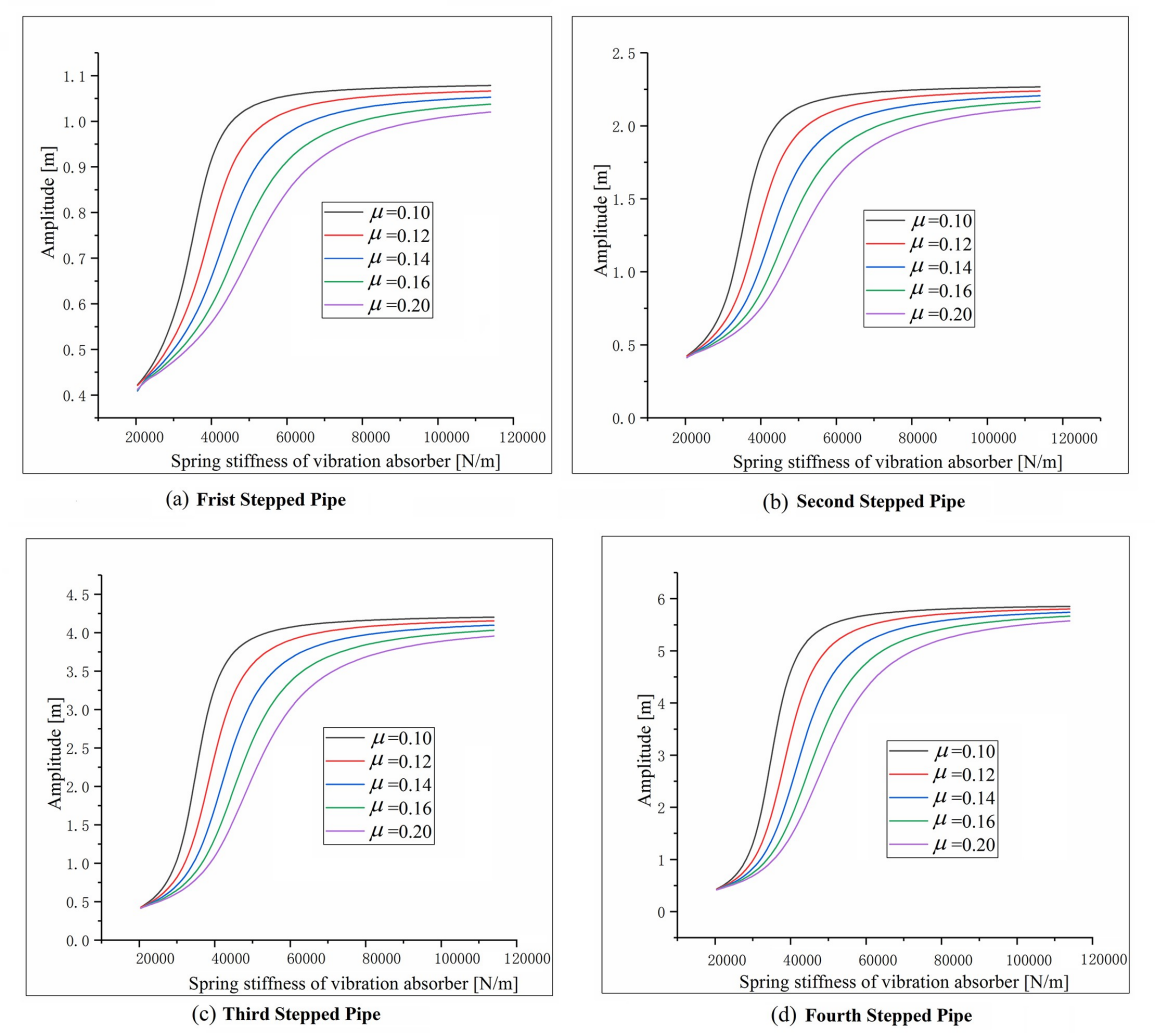
Amplitude comparison of stepped-pipe strings for different values of *k* and *μ*.

There is an optimum combination of the mass and spring constant for the dynamic vibration absorber to minimize the resonance amplitude and the maximum axial stress of the stepped-pipe string [8]. Fig 5 shows the amplitude of each stepped-pipe with different *μ* and *k* (spring stiffness constant).

It can be found that the change of *μ* value has little effect on vibration reduction if the value of spring stiffness is very small or large. We can obtain that the amplitude is smaller in case of the absorber with larger mass when the spring constant of dynamic vibration absorber varies between 0.2x10^5–^0.6x10^5^N/m.

**Fig 6:**
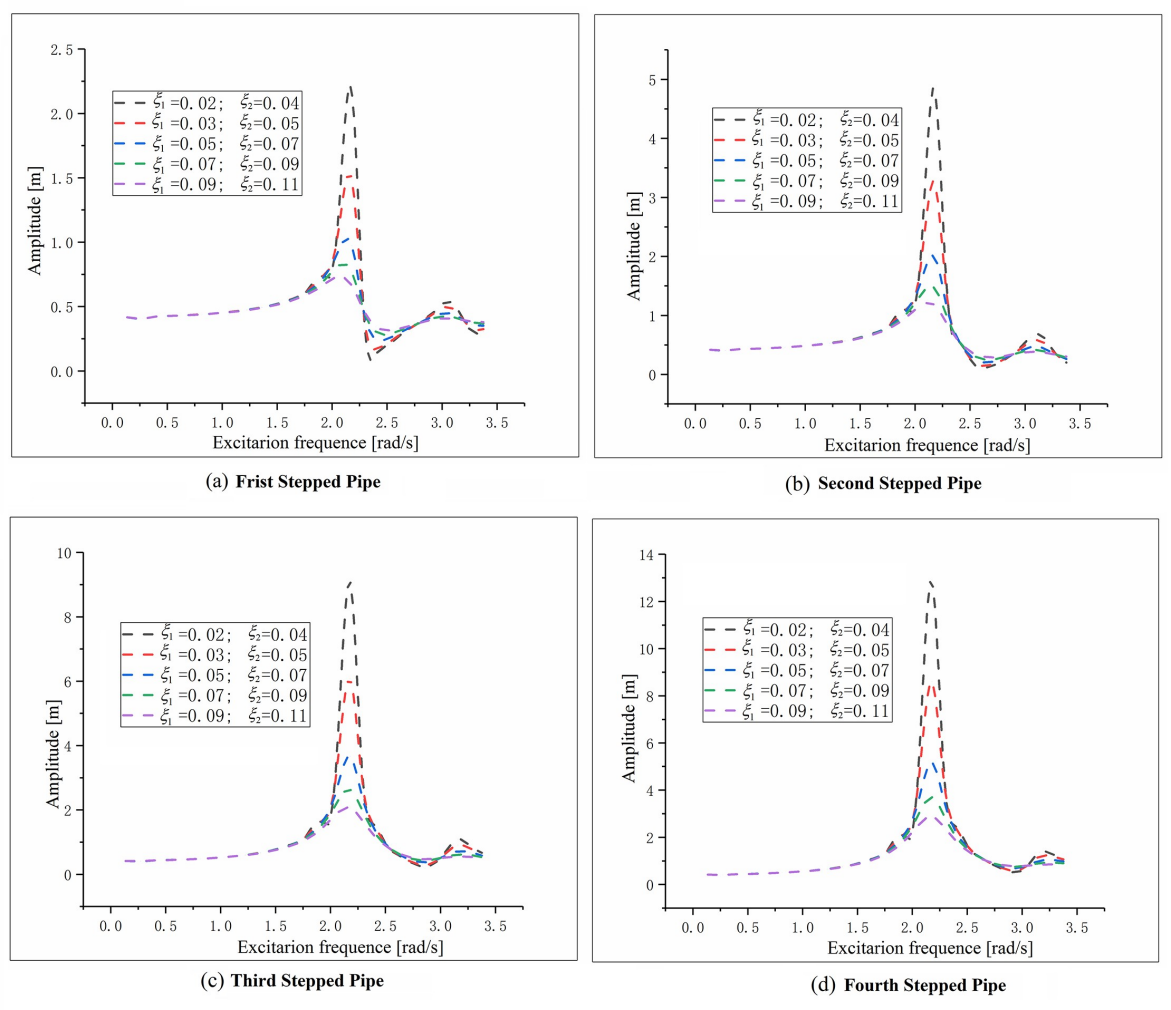
Dynamic response of stepped-pipe strings in different Rayleigh damping coefficients.

In addition, the longitudinal vibration response of the stepped-pipe strings system has also been compared for five Rayleigh damping coefficients (*ξ*). Fig 6 displays the comparison of *ξ* form 0.02 to 0.11.

It can be seen that the maximum value of vibration amplitude at different excitation frequencies decreases with the increase of damping coefficient *ξ*. In addition, The influence of *ξ* on the longitudinal vibration of the stepped-pipe strings increases first and then decreases, and the amplitude reaches the maximum when the excitation frequency is about 2.2rad/s. Fig 6 also indicates that the influence is the largest in the resonance region, but small in the non vibration region.

### 4.2 Analysis of inherent characteristics

**Fig 7:**
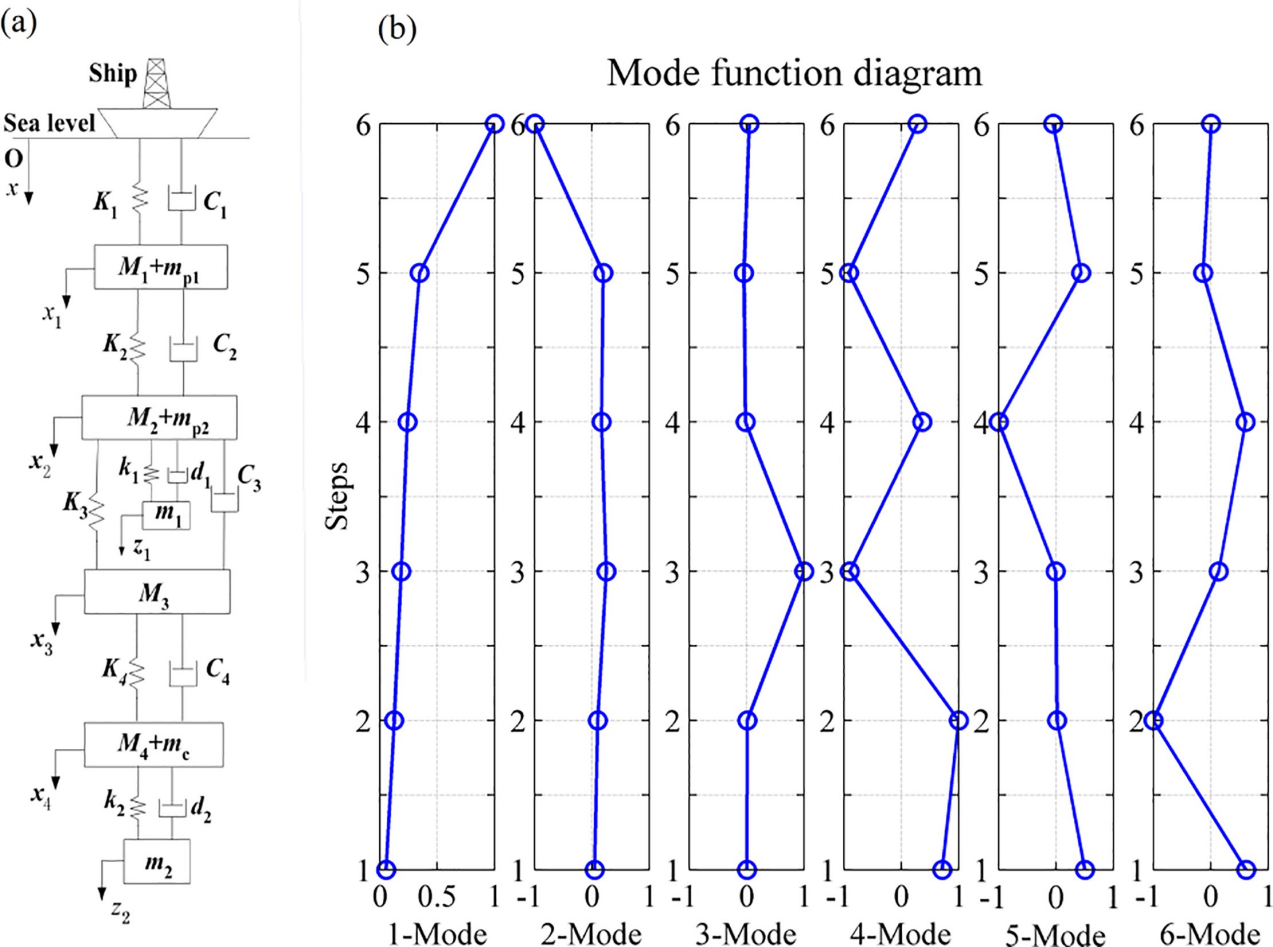
Schematic view of the system model and its first six mode shapes. (a) is the Schematic view of the model of the stepped-pipe strings with DVA. (b) is the modal figures of stepped-pipe strings with DVA.

The natural frequencies and modal vectors of the system were calculated by MATLAB, and the characteristic information is shown in Table 3. The first six orders vibration mode of stepped pipes are figured out, as shown in Fig 7.

**Table 3:** Characteristic information of principal modes with DVA.

Modal No.	Frequency (rad/s)	Maximum deformation (m)	Maximum deformation position
1	2.0860	4.3077	4-th stepped pipe
2	2.8249	1.4686	4-th stepped pipe
3	3.6329	0.7719	4-th stepped pipe
4	5.3042	0.9174	2-th stepped pipe
5	8.0147	0.3117	3-th stepped pipe
6	10.4167	0.2130	1-th stepped pipe

Fig 7(B) shows the mode shapes of the stepped-pipe strings with DVA, It can be seen that the mode shapes of the stepped-pipe strings can be determined using the equations and technique proposed in this paper. From Figs 3 and 7 it is observed that mode shapes obtained by attaching the vibration absorber and without attaching the vibration absorber are almost the same. However, it is obvious that the vibration amplitude with DVA is much smaller than that without DVA.

By comparing Tables 2 and 3, it could be concluded that the longitudinal vibration amplitude of the stepped-pipe strings can be greatly suppressed by attaching DVA to the buffer and second pump. Even it can make the amplitude of the primary system remain small in the whole-frequency range.

**Fig 8:**
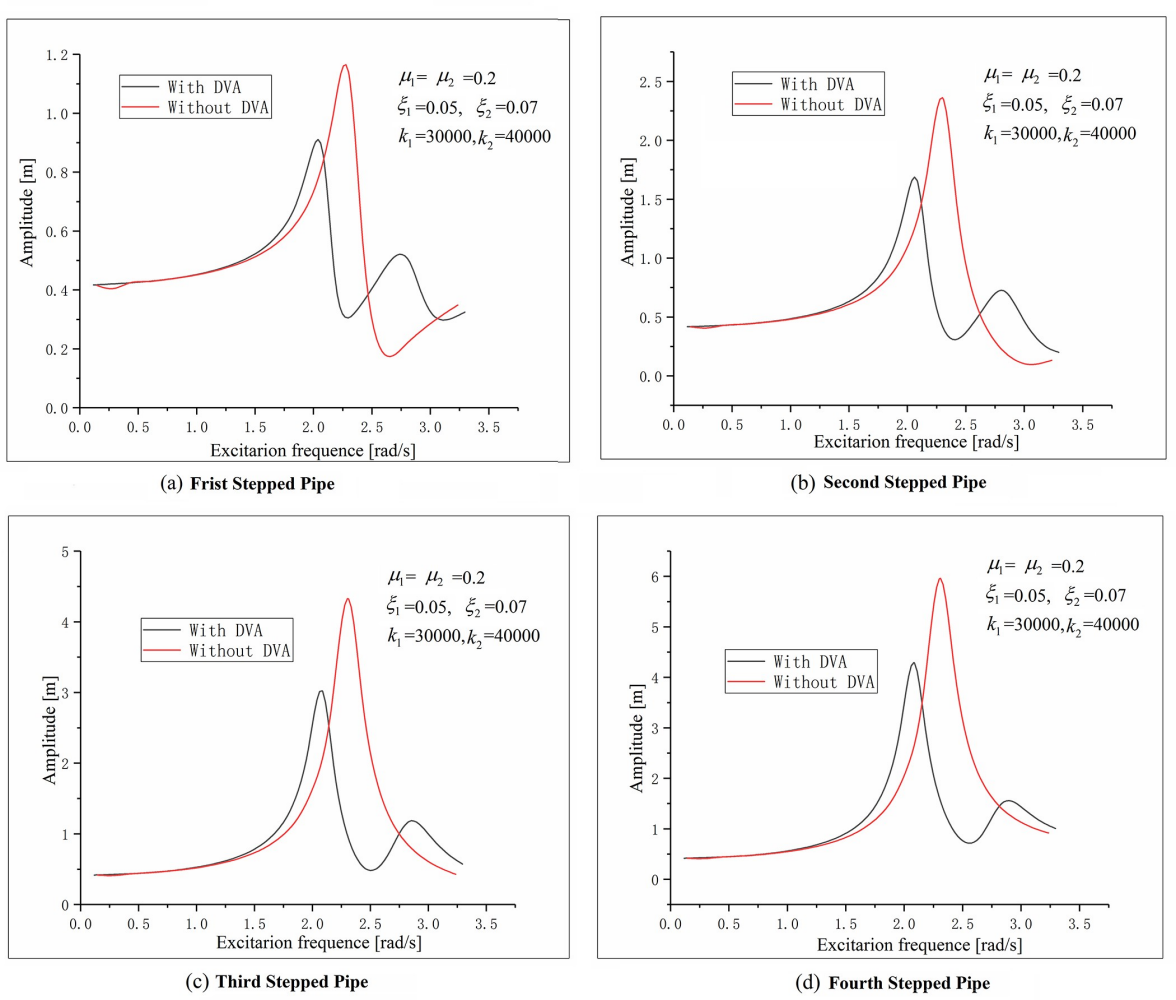
The amplitude frequency curves of different parts of stepped-pipe strings.

It is important to note the Fig 8, which is a result of the existence of an optimal combination of mass ratio(*μ*), spring coefficient (*k*) and damping ratio(*ξ*) near the first natural frequency.

The simulation results show that the maximum value of longitudinal vibration amplitude decreases sharply after attaching with dynamic vibration absorber at the pump and buffer. The maximum amplitude value of the second pump position decreases from 2.25*m* to 1.59*m*, and the maximum amplitude of buffer position decreases from 5.85*m* to 4.15*m*. And the natural frequency value changes from the original natural frequency to two natural frequency points.

From the above analysis, it can be seen that the longitudinal amplitude of the buffer at the bottom of the stepped-pipe strings is the largest, followed by the position of the second pump at the first order resonance. If the longitudinal amplitudes of these two positions are suppressed, the amplitudes of the whole stepped-pipe system will be improved correspondingly.

### 4.3 Calculation and analysis

The mining ship is simplified as a particle on the top of the stepped-pipe strings. The heave motion of the mining ship caused by wave fluctuation is simplified as simple harmonic motion, which produces an exciting force on the stepped-pipe strings. The exciting force was represented by a cosine wave excitation with the peak magnitude *F* of 12*k*N and the excitation frequency Ω of 2*rad*/*s*. The exciting force column vector can be expressed as:

$$\left\{f(t)\right\}={\left\{\begin{array}{cccccc} F\cos\Omega t & 0 & 0 & 0 & 0 & 0 \end{array}\right\}}^{T}$$ (24)

Therefore, the normalized modal generalized excitation force vector in the 6-degree-of-freedom stepped-pipe strings system is written as:

$$\left\{N(t)\right\}={\left[U_{N}\right]}^{T}\left\{f(t)\right\}={\left\{N_{1},N_{2},N_{3},N_{4},N_{5},N_{6}\right\}}^{T}\times \cos\Omega t$$ (25)

Using modal superposition method, Eq (23) is expanded as follows:

$${\ddot{q}}_{Ni}(t)+2\xi_{i}\omega_{ni}{\dot{q}}_{Ni}(t)+\omega^{2}{}_{ni}q_{Ni}(t)=N_{i}\cos\Omega t,i=1,2,3\ldots 6$$ (26)

Therefore, the output of forced longitudinal vibration of the stepped-pipe strings is determined by

$$q_{Nr}(t)=\sum_{i=1}^{6}\frac{F_{i}U_{Ni}^{\left(r\right)}\cos(\Omega_{i}t)}{\omega_{nr}^{2}-\Omega_{i}^{2}},\;r=1,2,\ldots ,6$$ (27)

**Fig 9:**
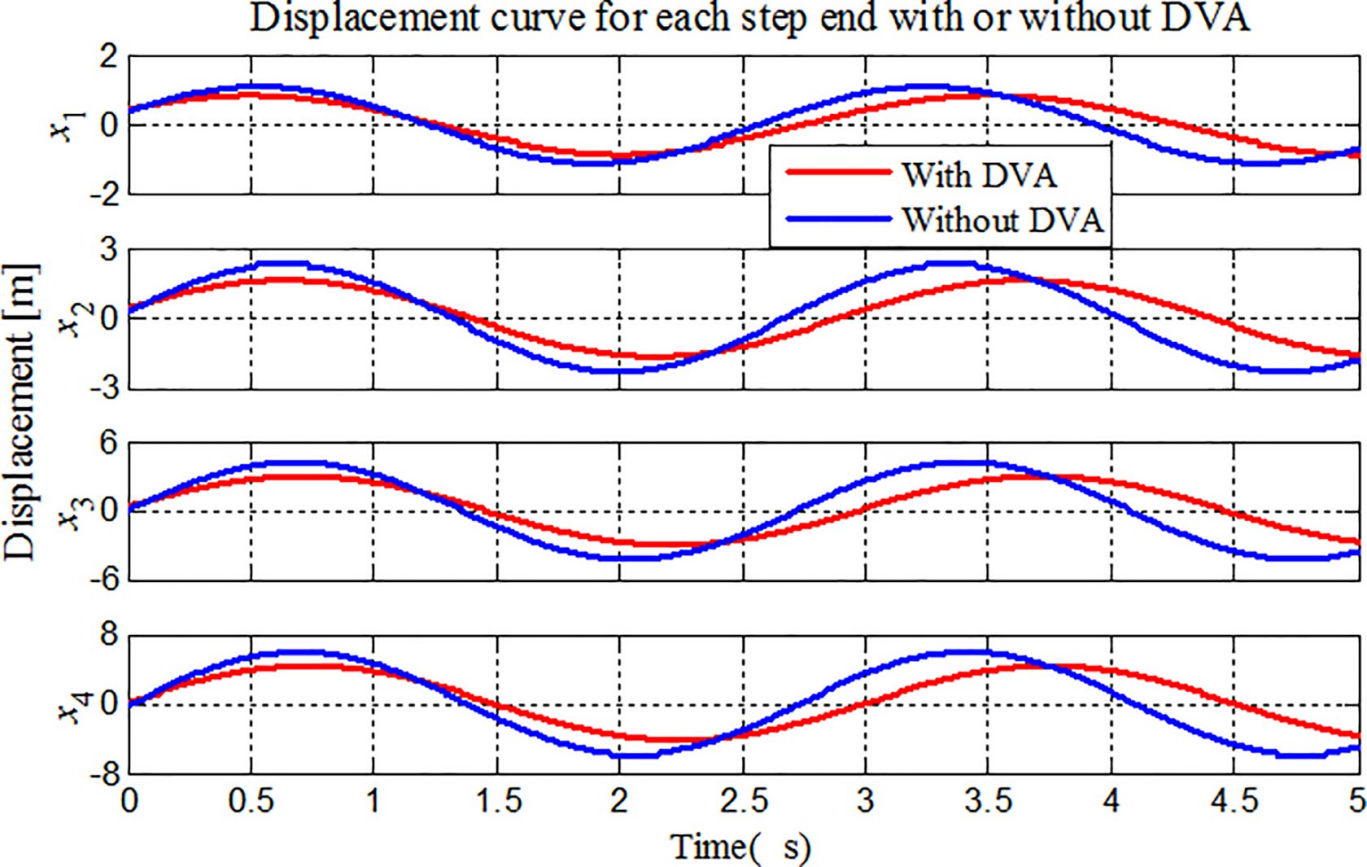
Displacement variations for each stepped-pipe end.

**Fig 10:**
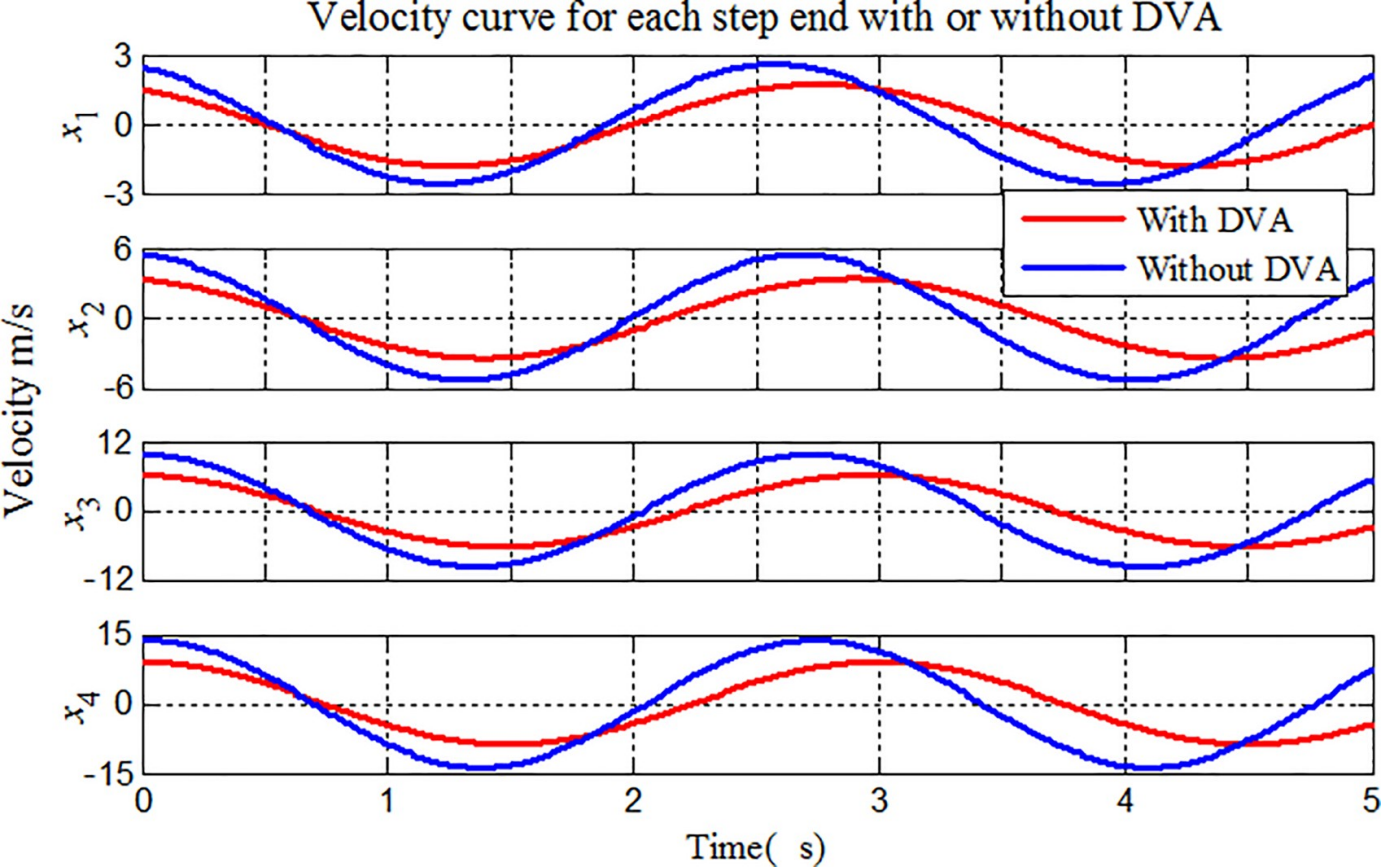
Velocity variations for each stepped-pipe end.

**Fig 11:**
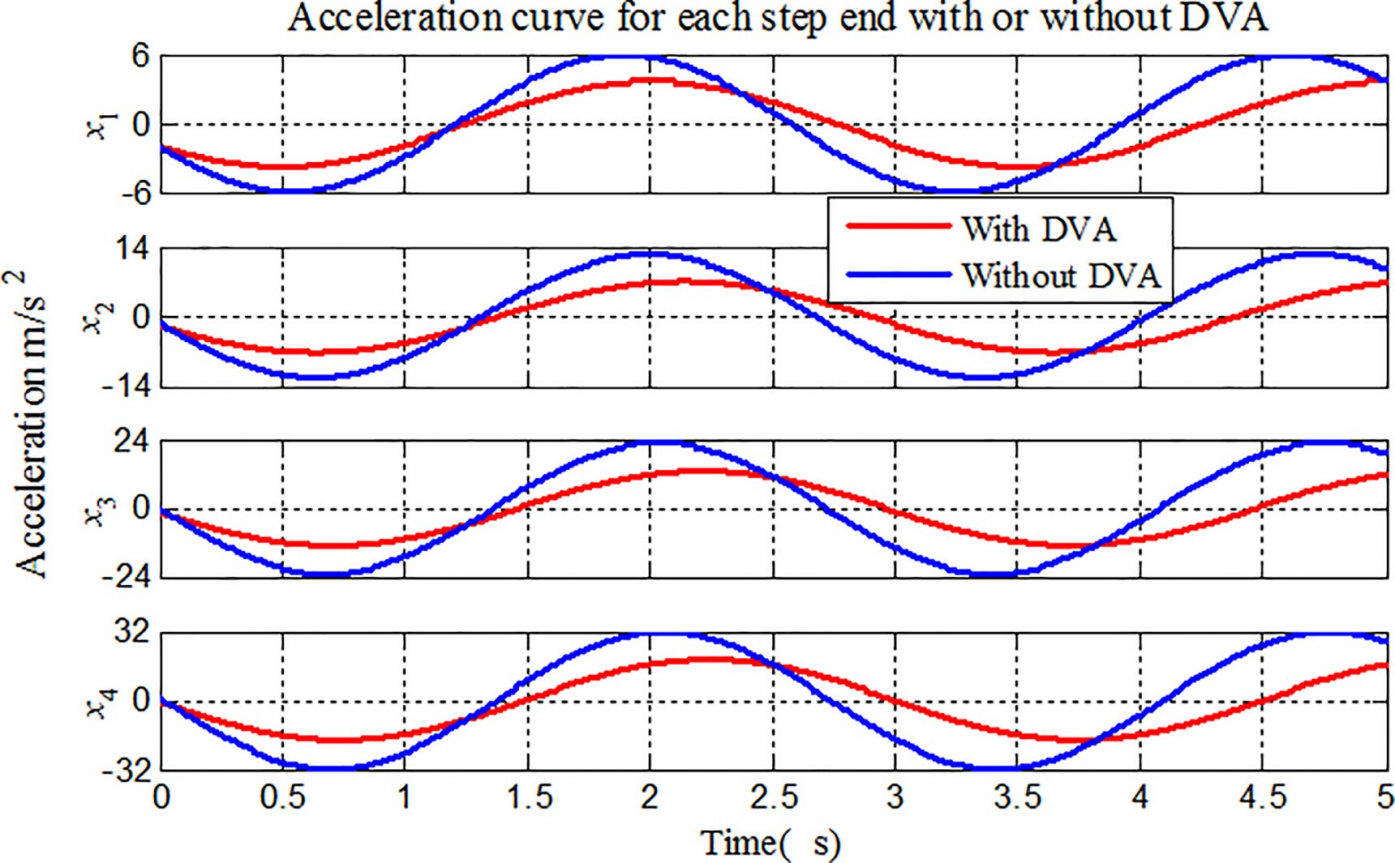
Acceleration variations for each stepped-pipe end.

To illustrate the application of the proposed method to the vibration analysis of stepped-pipe strings, we focus on the longitudinal displacement, velocity and acceleration of the stepped-pipe strings in Figs 9–11. The same previous simulation conditions are considered in the comparative analysis. During mining operation, the heave motion at the top of stepped-pipe strings is the sinusoidal harmonic movement with the angular frequency of 2*rad*/*s*.

In Figs 9–11, we have plotted the displacement, velocity and acceleration variations of longitudinal vibration at the bottom of each stepped-pipe when the stepped-pipe strings is subjected to sinusoidal movement at the top. In the same sea state, the amplitude of the lifting subsystem is gradually increased, that is, in the deep-sea mining operation, the vibration amplitude of each subsystem in the lifting system increases with the increase of the distance from the stepped-pipe strings to the sea level. On the other hand, the acceleration, velocity and displacement of the end of the stepped-pipe strings are greatly reduced after attaching DVAs at the end of the second and fourth stepped pipes respectively, and the vibration suppression effect can be reduced by 30%. It is thus clear that the attachment DVA on the stepped pipe is a simple, feasible and effective method to suppress the longitudinal vibration of stepped-pipe strings. It can be clearly seen from the figures that the theoretical calculation is in good agreement with the numerical results.

## 5 Conclusions

A unified and rigorous mass lumping scheme is proposed in this paper, which can be applicable to the longitudinal vibration analysis of stepped-pipe strings. Based on the Lagrangian equation and the background theory on vibration analysis of stepped-pipe strings for DSM, the multi-degrees-of-freedom dynamic model of the system was established. Modal testing has been carried out by sea breeze excitation. Out of the tested excitation, the stepped-pipe strings yields the accurate natural frequencies, and the good mode shapes are obtained. In the modal analysis of stepped-pipe strings without DVA, it is found that the vibration amplitude of the fourth part is the larges tin the first mode, followed by the second part in the second mode. After attaching DVA in the second and fourth parts, the vibration amplitude decreases obviously. The compensation rate [13] of the fourth part can reach more than 86%, and the compensation rate of the second part can reach more than 82%, so the proposed method had a better compensation effect.

Future research may take into account effects of other vibrations on the stepped-pipe strings and the parameters optimization of DVA in order to increase accuracy.

## Supporting information

S1 Data (TXT)

S2 Data (TXT)
